# Efficacy of ferulic acid in the treatment of acute ischemic stroke injury in rats: a systematic review and meta-analysis

**DOI:** 10.3389/fphar.2023.1278036

**Published:** 2023-10-19

**Authors:** Jiashan Wang, Meiqi Tang, Xiuzhen Xie, Yingqi Xu, Pingping Su, Zhuqing Jin

**Affiliations:** ^1^ The Third School of Clinical Medicine, Zhejiang Chinese Medical University, Hangzhou, China; ^2^ Department of Chemistry, Zhejiang University, Hangzhou, China; ^3^ The First School of Clinical Medicine, Zhejiang Chinese Medical University, Hangzhou, China; ^4^ The Second School of Clinical Medicine, Zhejiang Chinese Medical University, Hangzhou, China; ^5^ School of Basic Medicine Sciences, Zhejiang Chinese Medical University, Hangzhou, Zhejiang, China

**Keywords:** ferulic acid, acute ischemic stroke injury, excitotoxicity, inflammatory response, apoptosis, meta-analysis

## Abstract

**Background:** Intravenous thrombolysis is commonly used in the treatment of acute ischemic stroke damage. The existing thrombolytic drugs still suffer significant shortcomings, including a limited fibrin specificity and bleeding complications. Ferulic acid can directly bind the key thrombus enzymes and target to blood clots, suggesting its thrombolytic potency that may be beneficial with thrombolytic potency for the treatment of acute ischemic stroke damage.

**Objective:** The purpose of this systematic review and meta-analysis was to evaluate the efficacy of ferulic acid in the treatment of acute ischemic stroke injury in rats and its potential mechanism of action.

**Materials and methods:** We conducted a literature search in six databases, including CNKI, up to July 2023.

**Results:** Sixteen trials were included in the meta-analysis, which demonstrated that ferulic acid significantly reduced infarct size, neurological deficit score, apoptosis index, cleaved caspase-3, and cytochrome C levels (all *p* < 0.05). In addition, ferulic acid significantly increased the levels of phosphorylated Akt, mitochondrial Bcl-xL/Bax, phosphorylated astrocyte PEA15, hippocampal calcium binding protein, and mitochondrial Bcl-2/Bax ratio (all *p* < 0.05).

**Conclusion:** This study demonstrates that ferulic acid protects against acute ischemic stroke injury in rats by inhibiting ischemia-induced excitotoxicity, inflammatory response, and apoptosis.

## 1 Introduction

Ischemic stroke is a common condition in middle-aged and elderly individuals, particularly in those with underlying conditions like hypertension and heart disease (Kong and Zhu, 2017). If left untreated, neurological function can rapidly deteriorate within 6 hours of the onset of acute ischemic stroke (AIS), leading to aggravated symptoms and an increased likelihood of death (Fu and Song, 2022). Furthermore, survivors are often left with reduced mobility requiring assistance with daily living activities (Wu and Ning, 2016).

Intravenous thrombolysis is the primary treatment for AIS (Wang and Peng, 2022), which involves the administration of drugs such as alteplase and urokinase to promote fibrinolysis and dissolve blood clots. However, this method cannot accurately locate the site of bloakage or assess the severity of occlusive blood vessels, resulting in low vascular recirculation rates (Chen and Yang, 2022). It is often used in conjunction with other therapeutic approaches to achieve significant effects (Association NBoCM, Association CDGoNBoCM et al., 2022). Additionally, thrombolytic drugs can increase the risk of bleeding and reinfarction after treatment (Chen et al., 2022), which can have a detrimental effect on patient prognosis (Chinese Society of Neurology, Chinese Stroke Society. et al., 2019).

**FIGURE 1:**
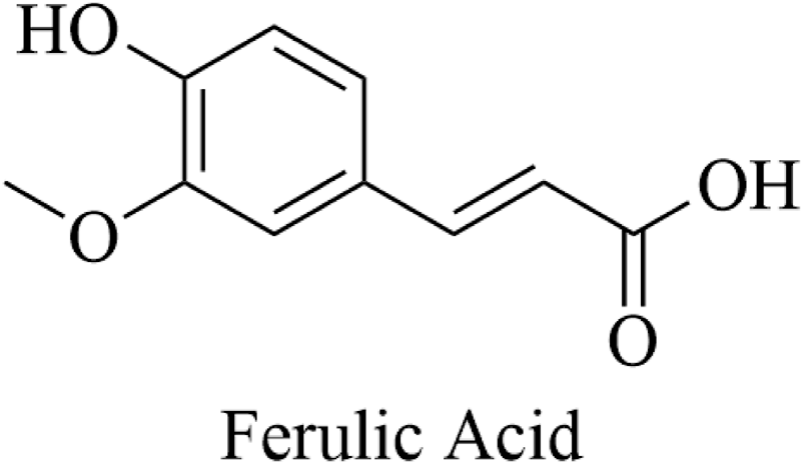
Chemical structures of ferulic acid.

Ferulic acid is the main active ingredient of *Angelica sinensis* (Zhang L. et al., 2016; Lv and Li, 2022), a commonly used traditional medicinal herb in China, which is derived from the dried root of the *Angelica Sinensis* Radix, an umbelliferae plant. It is often used to treat tumors, cancers, and a variety of cardiovascular and cerebrovascular diseases (Li and Ni, 2022). In Chinese Pharmacopoeia (2020 edition), *A. sinensis* only takes ferulic acid as the quality control index component, and its chemical structure is shown in Figure 1.

Ferulic acid, has been shown to have a prominent role in protecting and repairing damaged nerve cells and mitochondria as well as preventing microthrombosis by reducing fibrinogen deposition and inhibiting thrombin release and platelet aggregation (Wang and Wang, 2019). Moreover, ferulic acid can directly target the ischemic area by binding to key thrombus enzymes, thus providing timely protection to the affected nerve while exerting a significant therapeutic effect (Choi and Kim, 2018). Clinical studies have also demonstrated the safety and efficacy of ferulic acid in treating ischemic stroke, with a lower incidence of adverse effects compared to the conventional treatments (Zhou and Yang, 2012). However, the mechanisms underlying its action remain unclear.

Here we performed a meta-analysis in experimental animal models of ischemic stroke in rats to provide the potential mechanisms responsible for the cerebral protection by ferulic acid, which may further support the future clinical trial of ferulic acid in the treatment of ischemic shock in human.

## 2 Materials and methods

The meta-analysis was performed based on the PRISMA guidelines (Moher et al., 2009) and Cochrane Collaboration (Cumpston et al., 2019).

### 2.1 Literature retrieval strategy

The following databases were searched to identify relevant studies up to July 2023: Chinese National Knowledge Infrastructure (CNKI), Cqvip Database (VIP), Wanfang Data, Chinese Biomedical Database (SinoMed), Web of Science and PubMed. We identified the keywords “ ischemic stroke ” according to the latest Chinese Medicine thesaurus (MeSH) compiled by the US National Library of Medicine, and searched the database using the following keywords: for English databases, we used the search strategies as follows: ((ischemic stroke) OR (cerebral infarction) OR (Ischemic Encephalopathy) OR (Brain Ischemia) OR (Ischemic Strokes)) AND (ferulic acid); for Chinese databases, we used the search strategies as follows: SU = “Nao Que Xue” + “Nao Geng Si” + “Que Xue Xing Cu Zhong” + “Que Xue Xing Nao Geng Si” + “Que Xue Xing Zhong Feng” AND SU = “A Wei Acid”.

### 2.2 Literature screening criteria

#### 2.2.1 Research subject

Permanent ischemic stroke or ischemic stroke/reperfusion models in male Sprague-Dawley rats.

#### 2.2.2 Type of study

Trials, no restrictions on language and time.

#### 2.2.3 Interventions

The experimental group was ischemic stroke model with ferulic acid injection, and the control group was ischemic stroke model only without ferulic acid injection.

#### 2.2.4 Outcome measures

Apoptosis index was calculated as the percentage of TUNEL positive cells in the total number of cells during ischemic stroke. In this study, the indicators were divided into primary outcome indicators and secondary outcome indicators according to whether they could directly reflect the efficacy of ferulic acid in the treatment of AIS animal models. The primary outcome indicators included infarct size and neurological deficit score, which could most directly reflect the brain protective effect of ferulic acid on AIS injury. Secondary outcome indicators were divided into excitotoxicity marker (hippocampal calcium-binding protein level), inflammatory response marker (phosphorylated astrocyte PEA-15 level), apoptotic index and apoptotic markers (mitochondrial Bcl-xL/Bax, levels of phosphorylated Akt, mitochondrial Bcl-2/Bax, cytochrome c level, cleaved caspase-3 level).

#### 2.2.5 Exclusion criteria

1) Repeated and irrelevant literature; 2) Non-rat ischemic stroke models; 3) Drug combination therapy; 4) Literature with incomplete content and missing data; 5) Failure to meet the outcome indicators or failure to extract effective indicators; 6) Reviews, conferences, cases, dissertations, and clinical studies.

#### 2.2.6 Literature screening and data extraction

The literature was independently searched by two professionally trained researchers, and the results were uniformly imported into NoteExpress software. If any disputes arose, a third researcher participated in the discussion and cross-review to determine the final inclusion of the literature. Results were summarized in a data collection table that included the title, first author, year of publication, country, model, modeling method, ferulic acid dose, administration method, intervention measures, course of treatment, and outcome index. In cases where literature reported data only in the form of images, we utilized GetData Graph Digitizer to extract the data.

#### 2.2.7 Literature quality assessment

This study used an examination scale (Aliena-Valero et al., 2021) with 15 evaluation criteria that combined the first 10-item CAMARADES’s checklist (Sena et al., 2007) and the updated STAIR criteria (Fisher et al., 2009). Two researchers independently used the scale to assess the quality of each item according to the criteria as follows: 1) peer-reviewed publication, 2) control of temperature, 3) randomisation of group allocation, 4) blinded induction of ischaemia, 5) blinded assessment of outcome, 6) avoidance of anaesthetics with marked intrinsic neuroprotective properties, 7) use of animals with co-morbidities (e.g., hypertension, diabetes), 8) sample size calculation, 9) statement of compliance with animal welfare requirements, 10) statement of potential conflicts of interest, 11) physiological monitoring during stroke induction (in addition to control of temperature, e.g., blood pressure, gases), 12) prespecified inclusion and exclusion criteria, 13) reporting of animals excluded from analysis, 14) reporting of study funding and 15) injury confirmed via laser Doppler or perfusion imaging.

#### 2.2.8 Statistical analysis

Revman 5.4 software was used for statistical analysis of at least two studies reporting the same outcome measures. All outcomes were continuous variables except neurological deficit score, which was classified ordinal variable. The mean difference and 95% confidence interval (CI) were used to estimate the effect of ferulic acid. I^2^ was used to test the heterogeneity of the results among studies. If the heterogeneity was low (I^2^<50%), the fixed-effect model was used for meta-analysis. If the heterogeneity was high (I^2^ ≥ 50%), the random-effects model was used for meta-analysis, and sensitivity and subgroup analyses were conducted to explore the sources of heterogeneity. The final results were presented in forest plots and funnel plots.

## 3 Result

### 3.1 Included literature

A total of 1048 relevant literature were retrieved. Firstly, 508 duplicate literature were removed using NoteExpress software.

**FIGURE 2:**
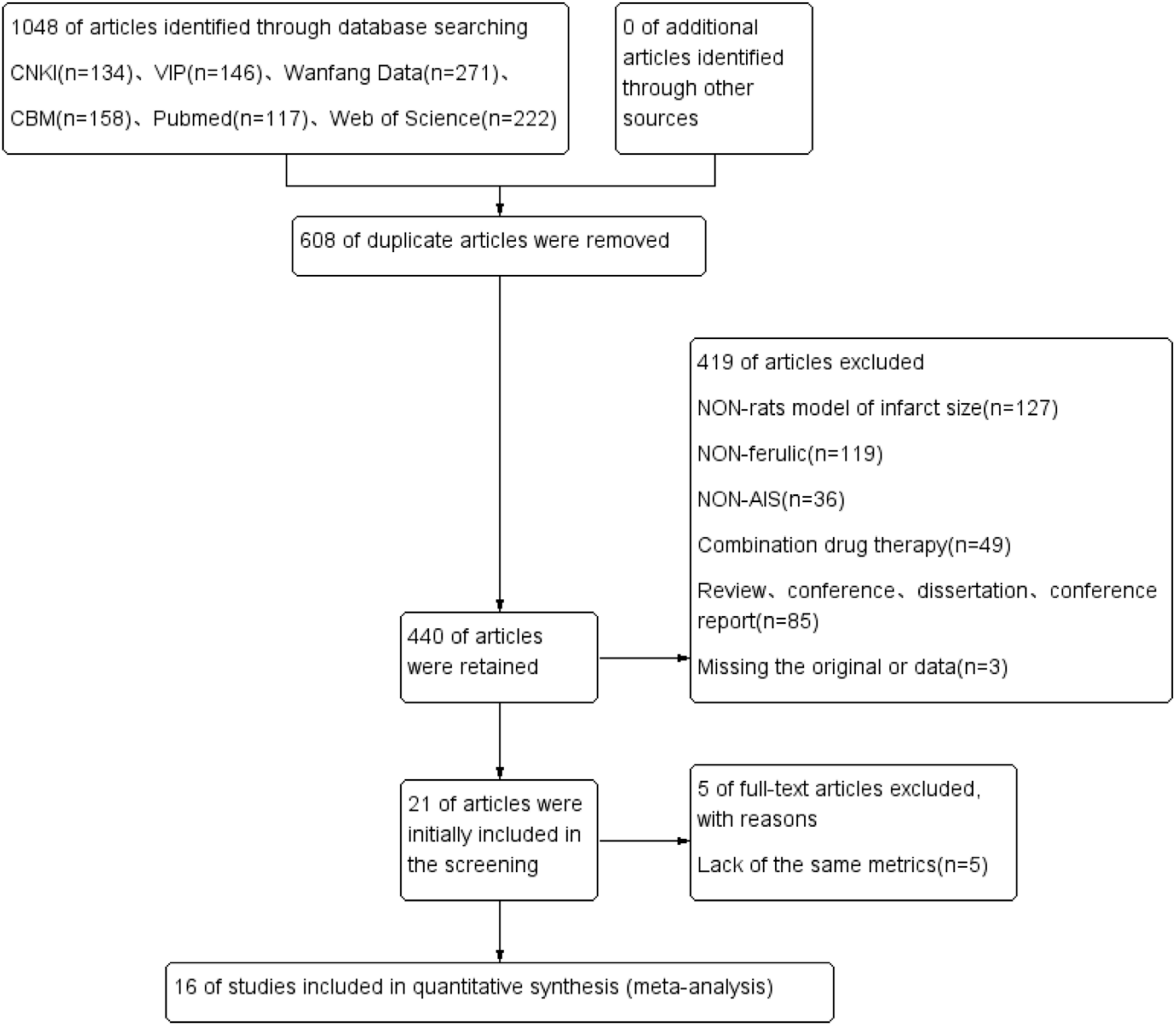
Literature search flow chart.

In the next step, the abstracts and full texts were carefully read, and studies were excluded if they had incomplete content, missing data, used non-rat models of ischemic stroke, were reviews, conference papers, case reports, dissertations, clinical studies, or involved combined drug therapy. As a result, 21 studies were obtained. Of those, 5 studies that did not conform to the outcome indicators or could not provide effective indicators were excluded, and 16 studies were included in the final meta-analysis (Figure 2).

### 3.2 Study characteristics

In this study, a total of 16 studies were included, 13 of which were from different groups of researchers. All of which used male SD rats with body weights ranging from 200g to 350 g. Among these studies, 11 used the permanent ischemic stroke model, while 5 studies used the ischemic stroke/reperfusion model. For preparation of the middle cerebral artery occlusion model, 15 studies used the intraluminal filament model and 1 study used the vascular clipping model. The details of the included studies are presented in Table 1, while information about ferulic acid used in these studies is shown in Table 2.

**TABLE 1:** Basic characteristics of the study literature are included.

Study	Country	Animal characteristics	Model	Modeling methods	Ferulic acid dosage (mg/kg)	Method of administration	Interventions		Course	Outcome measures
							T	C		
Sung et al. (2012)	Korea	Male SD rats (220–230 g)	Permanent ischemic stroke	Wire bolt method	100	intravenous injection	Ferulic acid	Vehicle	24 h	⑦⑧
Zhong et al. (2017)	China	Male SD rats (220 ± 20 g)	Ischemic stroke -reperfusion	Vascular occlusion	56	intravenous injection	Ferulic acid	Vehicle	5 d	④
Koh (2013a)	Korea	Male SD rats (210–220 g)	Permanent ischemic stroke	Wire bolt method	100	intravenous injection	Ferulic acid	Vehicle	24 h	①
Koh (2012a)	Korea	Male SD rats (225–230 g)	Permanent ischemic stroke	Wire bolt method	100	intravenous injection	Ferulic acid	Vehicle	24 h	①④⑤
Koh (2013b)	Korea	Male SD rats (225–250 g)	Permanent ischemic stroke	Wire bolt method	100	intravenous injection	Ferulic acid	Vehicle	24 h	①
Koh (2015)	Korea	Male SD rats (220–230 g)	Permanent ischemic stroke	Wire bolt method	100	intravenous injection	Ferulic acid	Vehicle	24 h	①
Koh (2013c)	Korea	Male SD rats (210–230 g)	Permanent ischemic stroke	Wire bolt method	100	intravenous injection	Ferulic acid	Vehicle	24 h	①⑧
Koh (2012b)	Korea	Male SD rats (210–230 g)	Permanent ischemic stroke	Wire bolt method	100	intravenous injection	Ferulic acid	Vehicle	24 h	⑦
Gim et al. (2013)	Korea	Male SD rats (210–230 g)	Permanent ischemic stroke	Wire bolt method	100	intravenous injection	Ferulic acid	Vehicle	24 h	⑤
Cheng et al. (2008a)	China	Male SD rats (300–350 g)	Ischemic stroke -reperfusion model	Wire bolt method	100	intravenous injection	Ferulic acid	Vehicle	24 h	①
Cheng et al. (2019a)	China	Male SD rats (300–350 g)	Permanent ischemic stroke	Wire bolt method	100	intravenous injection	Ferulic acid	Vehicle	7 d	①②③④⑥⑨⑩
Cheng et al. (2016)	China	Male SD rats (300–350 g)	Ischemic stroke -reperfusion model	Wire bolt method	100	intravenous injection	Ferulic acid	Vehicle	7 d	①③④⑥⑨⑩
Cheng et al. (2010)	China	Male SD rats (300–350 g)	Ischemic stroke -reperfusion model	Wire bolt method	100	intravenous injection	Ferulic acid	Vehicle	24 h	①④
Cheng et al. (2019b)	China	Male SD rats (300–350 g)	Permanent ischemic stroke	Wire bolt method	100	intravenous injection	Ferulic acid	Vehicle	3 d	①③④⑥⑨⑩
Cheng et al., 2008b	China	Male SD rats (300–350 g)	Ischemic stroke -reperfusion model	Wire bolt method	100	intravenous injection	Ferulic acid	Vehicle	24 h	①

SD, sprague dawley; T, experimental group; C, control group.

**Notes:**①infarct size ②apoptosis index ③neurological deficit score ④cleaved caspase-3, levels ⑤phosphorylated Akt levels ⑥mitochondrial Bcl-xL/Bax ⑦phosphorylated astrocyte PEA15 levels ⑧hippocampal calcium binding protein levels ⑨mitochondrial Bcl-2/Bax ⑩cytochrome C levels.

**TABLE 2:** Summary of ferulic acid used in the included studies.

Study	Source	Compound	Purity (%)	Quality control reported
		Concentration		
Sung et al. (2012)	Sigma, St. Louis, MO, United States	FA, NR	≥99.0%	HPLC
Zhong et al. (2017)	Sigma, St. Louis, MO, United States	FA, NR	≥99.0%	HPLC
Koh (2013a)	Sigma, St. Louis, MO, United States	FA, NR	≥99.0%	HPLC
Koh (2012a)	Sigma, St. Louis, MO, United States	FA, NR	≥99.0%	HPLC
Koh (2013b)	Sigma, St. Louis, MO, United States	FA, NR	≥99.0%	HPLC
Koh (2015)	Sigma, St. Louis, MO, United States	FA, NR	≥99.0%	HPLC
Koh (2013c)	Sigma, St. Louis, MO, United States	FA, NR	≥99.0%	HPLC
Koh (2012b)	Sigma, St. Louis, MO, United States of America.	FA, NR	≥99.0%	HPLC
Gim et al. (2013)	Sigma, St. Louis, MO, United States	FA, NR	≥99.0%	HPLC
Cheng et al. (2008a)	Sigma, St. Louis, MO, United States	FA, NR	≥99.0%	HPLC
Cheng et al. (2019a)	Sigma, St. Louis, MO, United States	FA, NR	≥99.0%	HPLC
Cheng et al. (2016)	Sigma, St. Louis, MO, United States	FA, NR	≥99.0%	HPLC
Cheng et al. (2010)	Sigma, St. Louis, MO, United States	FA, NR	≥99.0%	HPLC
Cheng et al. (2019b)	Sigma, St. Louis, MO, United States	FA, NR	≥99.0%	HPLC
Cheng et al., 2008b	Sigma, St. Louis, MO, United States	FA, NR	≥99.0%	HPLC

FA, ferulic acid; NR, no report; HPLC, high performance liquid chromatography.

### 3.3 Risk of bias assessment

In this study, only 1 study was not explicitly declared to be randomized or nonrandomized. 3 studies mentioned the blinding of investigators. None of the studies mentioned the use of blinding methods to induce cerebral ischemia in rats and the use of animals with comorbidities; 1 study did not avoid the use of anesthetics with significant intrinsic neuroprotective properties. 3 studies mentioned physiological monitoring such as blood pressure and gas during stroke induction. 3 studies did not confirm injury by laser Doppler or perfusion imaging. The bias risk of the specific included literature is shown in Table 3.

**TABLE 3:** Study quality report.

Study	Country	1	2	3	4	5	6	7	8	9	10	QS (0–10)	11	12	13	14	15	QS (0–15)
Sung et al. (2012)	Korea	+	+	+			+		+	+		6				+	+	8
Zhong et al. (2017)	China	+		+					+	+		4				+	+	6
Koh (2013a)	Korea	+	+	+			+		+	+		6					+	7
Koh (2012a)	Korea	+	+	+			+		+	+		6				+	+	8
Koh (2013b)	Korea	+	+	+			+		+	+		6				+	+	8
Koh (2015)	Korea	+	+	+			+		+	+		6				+	+	8
Koh (2013c)	Korea	+		+			+		+	+		5				+	+	7
Koh (2012b)	Korea	+					+		+	+		4				+		5
Gim et al. (2013)	Korea	+	+	+			+		+	+		6				+		7
Cheng et al. (2008a)	Korea	+	+	+			+		+	+		6				+		7
Cheng et al. (2019a)	China	+	+	+			+		+	+		6	+			+	+	9
Cheng et al. (2016)	China	+	+	+		+	+		+	+	+	8				+	+	10
Cheng et al. (2010)	China	+	+	+			+		+	+		6				+	+	8
Cheng et al. (2019b)	China	+	+	+			+		+	+		6	+			+	+	9
Cheng et al., 2008b	China	+	+	+		+	+		+	+		7				+	+	9
Sung et al. (2012)	China	+	+	+		+	+		+	+		7	+			+	+	10

**Notes:** (1) peer-reviewed publication, (2) control of temperature, (3) randomisation of group allocation, (4) blinded induction of ischaemia, (5) blinded assessment of outcome, (6) avoidance of anaesthetics with marked intrinsic neuroprotective properties, (7) use of animals with co-morbidities (e.g., hypertension, diabetes), (8) sample size calculation, (9) statement of compliance with animal welfare requirements, (10) statement of potential conflicts of interest, (11) physiological monitoring during stroke induction (in addition to control of temperature, e.g., blood pressure, gases), (12) prespecified inclusion and exclusion criteria, (13) reporting of animals excluded from analysis, (14) reporting of study funding and (15) injury confirmed via laser Doppler or perfusion imaging.

### 3.4 Effects of interventions

#### 3.4.1 Infarct size

**FIGURE 3:**
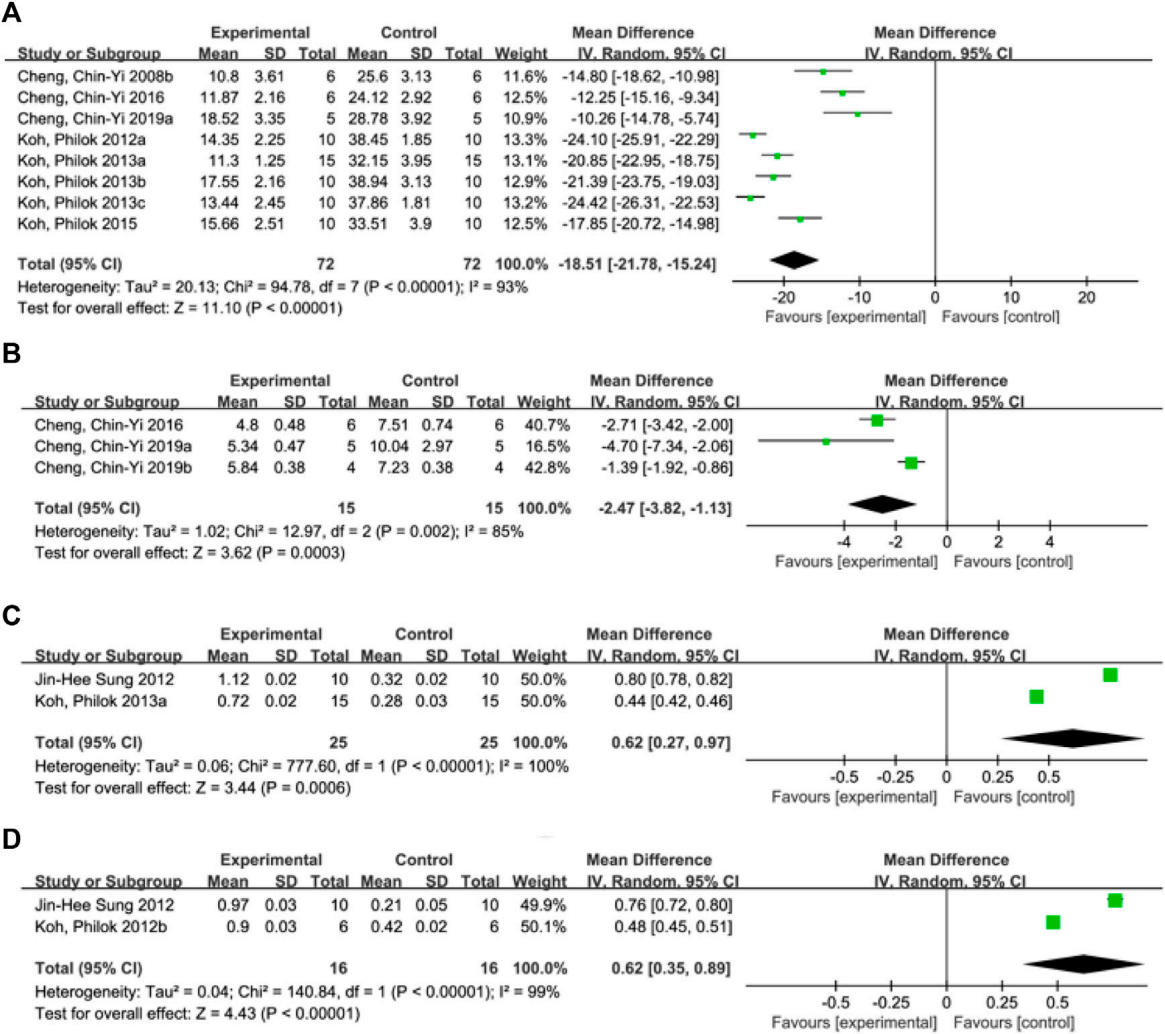
Forest plot of infarct size **(A)**, neurological deficit score **(B)**, hippocampal calcium binding protein levels **(C)** and phosphorylated astrocyte PEA15 levels **(D)**.

A total of 8 studies (Cheng et al., 2008b; Koh, 2012a; Koh, 2013a; Koh, 2013b; Koh, 2013c; Koh, 2015; Cheng et al., 2016; Chen et al., 2019) reported the infarct size. Random effect model was used because of the heterogeneity of the studies (*p* < 0.00001, I^2^ = 93%). The results showed that MD = −18.51, 95%CI = [−21.78, −15.24], *p* < 0.00001, indicating that ferulic acid could significantly reduce infarct size, and the difference was statistically significant. (Figure 3A).

#### 3.4.2 Neurological deficit score

A total of three studies (Cheng et al., 2016; Cheng et al., 2019a; Cheng et al., 2019b) reported neurological deficit score, and a random-effects model was adopted due to the high heterogeneity of the studies (*p* = 0.002, I^2^ = 85%). The results showed that the mean difference (MD) was −2.47, with a 95% CI of [−3.82, −1.13] and a *p*-value of 0.0003. This suggests that ferulic acid can reduce neurological deficit score of AIS, and the difference was statistically significant (Figure 3B).

#### 3.4.3 Hippocampal calcium binding protein levels

Two studies (Sung et al., 2012; Koh, 2013c) reported hippocampal calcium binding protein levels, and a random-effects model was adopted due to high heterogeneity (*p* < 0.00001, I^2^ = 100%). The results showed that ferulic acid had a significant effect on the level of hippocampal calcium-binding protein in focal ischemic stroke, with a mean difference (MD) of 0.62 (95% CI: 0.27, 0.97; *p* = 0.0006). The difference was statistically significant. (Figure 3C).

#### 3.4.4 Phosphorylated astrocyte PEA15 levels

A total of two studies (Koh, 2012b; Sung et al., 2012) reported the level of phosphorylated astrocyte PEA15. Due to the high heterogeneity of the studies (*p* < 0.00001, I2 = 99%), a random effects model was used. The results showed that the mean difference (MD) was 0.62 (95% CI: 0.35,0.89; *p* < 0.00001), indicating that ferulic acid had a significant effect on improving the level of phosphorylated astrocyte PEA15 during AIS. The difference was statistically significant (Figure 3D).

#### 3.4.5 Apoptosis index

**FIGURE 4:**
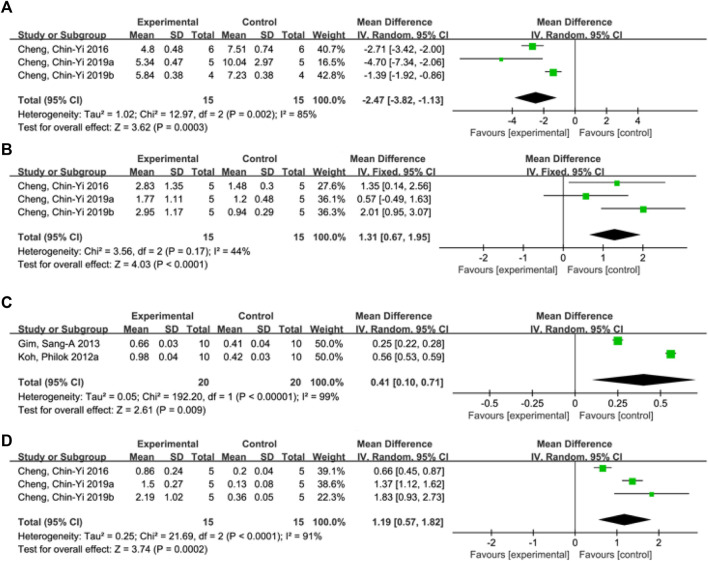
Forest plots of apoptosis index **(A)**, mitochondrial Bcl-xL/Bax **(B)**, phosphorylated Akt level **(C)** and mitochondrial Bcl-2/Bax **(D)**.

A total of 4 studies (Cheng et al., 2008a; Cheng et al., 2010; Cheng et al., 2019a; Cheng et al., 2019b) reported apoptosis index, and random effects model was adopted due to the high heterogeneity of the studies (*p* < 0.00001, I^2^ = 98%). The results showed that MD = −229.85, 95%CI = [−428.64, −31.07], *p* = 0.02, suggesting that ferulic acid can reduce the apoptosis index in the AIS, and the difference was statistically significant. (Figure 4A).

#### 3.4.6 Mitochondrial Bcl-xL/Bax

A total of three studies (Cheng et al., 2016; Cheng et al., 2019a; Cheng et al., 2019b) reported mitochondrial Bcl-xL/Bax levels, and a fixed-effects model was adopted due to low heterogeneity among the studies (*p* = 0.17, I^2^ = 44%). The results showed that the mean difference (MD) was 1.31, with a 95% CI of [0.67, 1.95], and *p* < 0.0001, indicating that ferulic acid could increase the ratio of mitochondrial Bcl-xL to Bax, and the difference was statistically significant (Figure 4B).

#### 3.4.7 Phosphorylated Akt level

A total of two studies (Koh, 2012a; Gim et al., 2013) reported the level of phosphorylated Akt, and a random effects model was used due to high heterogeneity (*p* < 0.00001, I^2^ = 99%). The results showed that the mean difference (MD) was 0.41 with a 95%CI of [0.10, 0.71] and a *p*-value of 0.009. These findings suggest that ferulic acid had a significant effect on phosphorylated Akt, and the difference was statistically significant (Figure 4C).

#### 3.4.8 Mitochondrial Bcl-2/Bax

A total of three studies (Cheng et al., 2016; Cheng et al., 2019a; Cheng et al., 2019b) reported mitochondrial Bcl-2/Bax, and random effects model was adopted due to the high heterogeneity of all studies (*p* < 0.00001, I^2^ = 97%). The results showed that ferulic acid group could increase the ratio of mitochondrial Bcl-2 to Bax, and the difference was statistically significant (MD = 1.19, 95%CI = [0.57, 1.82], *p* = 0.0002). (Figure 4D).

#### 3.4.9 Cytochrome C levels

**FIGURE 5:**
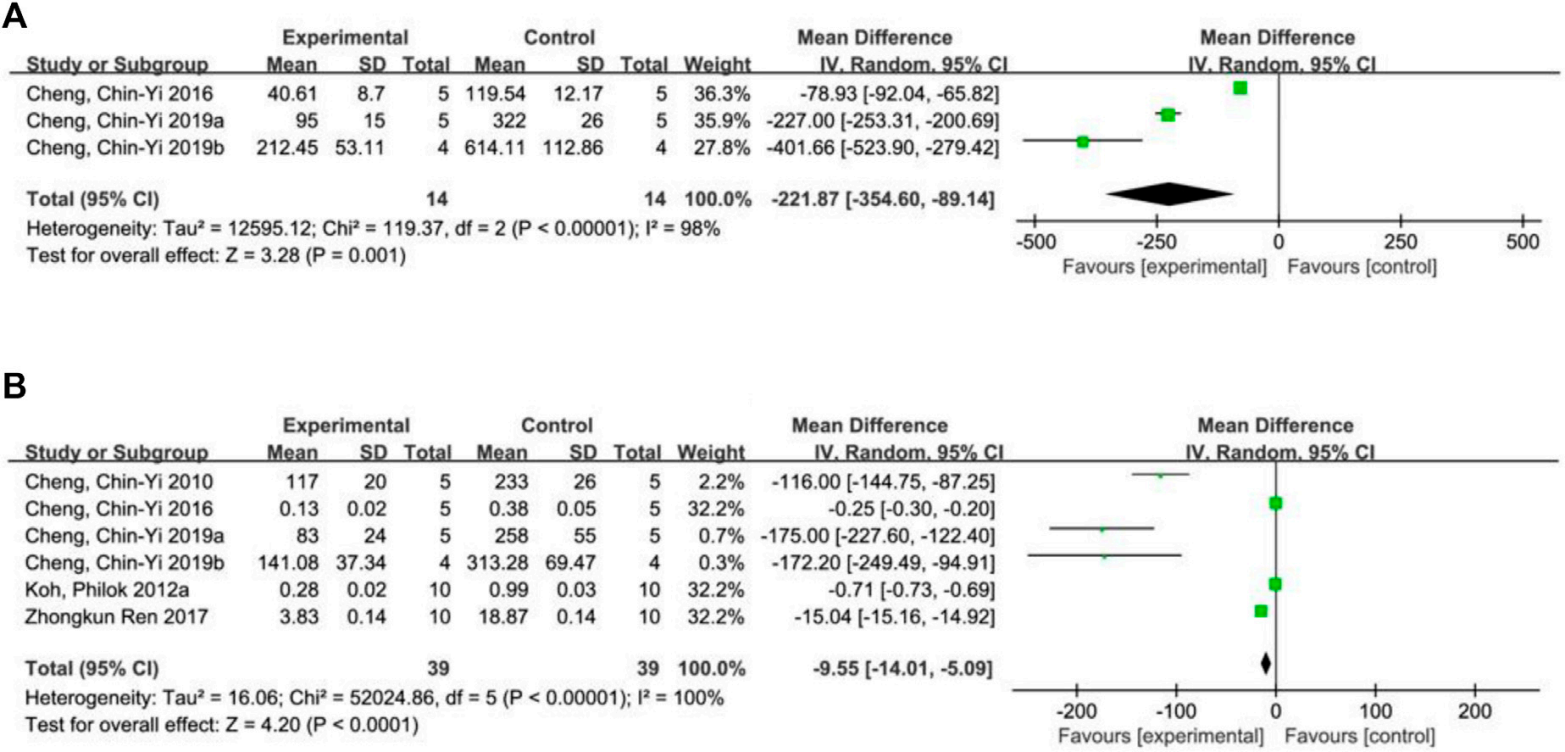
Forest plot of cytochrome C level **(A)**and cleaved caspase-3 horizontal **(B)**.

A total of 3 studies (Cheng et al., 2016; Cheng et al., 2019a; Cheng et al., 2019b) reported cytochrome C levels, and random effects model was adopted due to the high heterogeneity of all studies (*p* < 0.00001, I^2^ = 98%). The results showed that ferulic acid significantly decreased cytochrome C level during ischemic stroke (MD = −221.87, 95%CI = [−354.60, −89.14], *p* = 0.001) (Figure 5A).

#### 3.4.10 Cleaved Caspase-3 level

Six studies (Zhong et al., 2017; Koh, 2012a; Cheng et al., 2010; Cheng et al., 2016; Cheng et al., 2019a; Cheng et al., 2019b) reported cleaved caspase-3 levels, and a random-effects model was used due to the high heterogeneity of all studies (*p* < 0.00001, I^2^ = 100%). The results showed that the mean difference (MD) was −9.55, with a 95% CI of [−14.01, −5.09] and a *p*-value of <0.0001, indicating that the ferulic acid group could effectively reduce cleaved caspase-3 levels, and the difference was statistically significant (Figure 5B).

#### 3.4.11 Sensitivity analysis

##### 3.4.11.1 Infarct size

**FIGURE 6:**
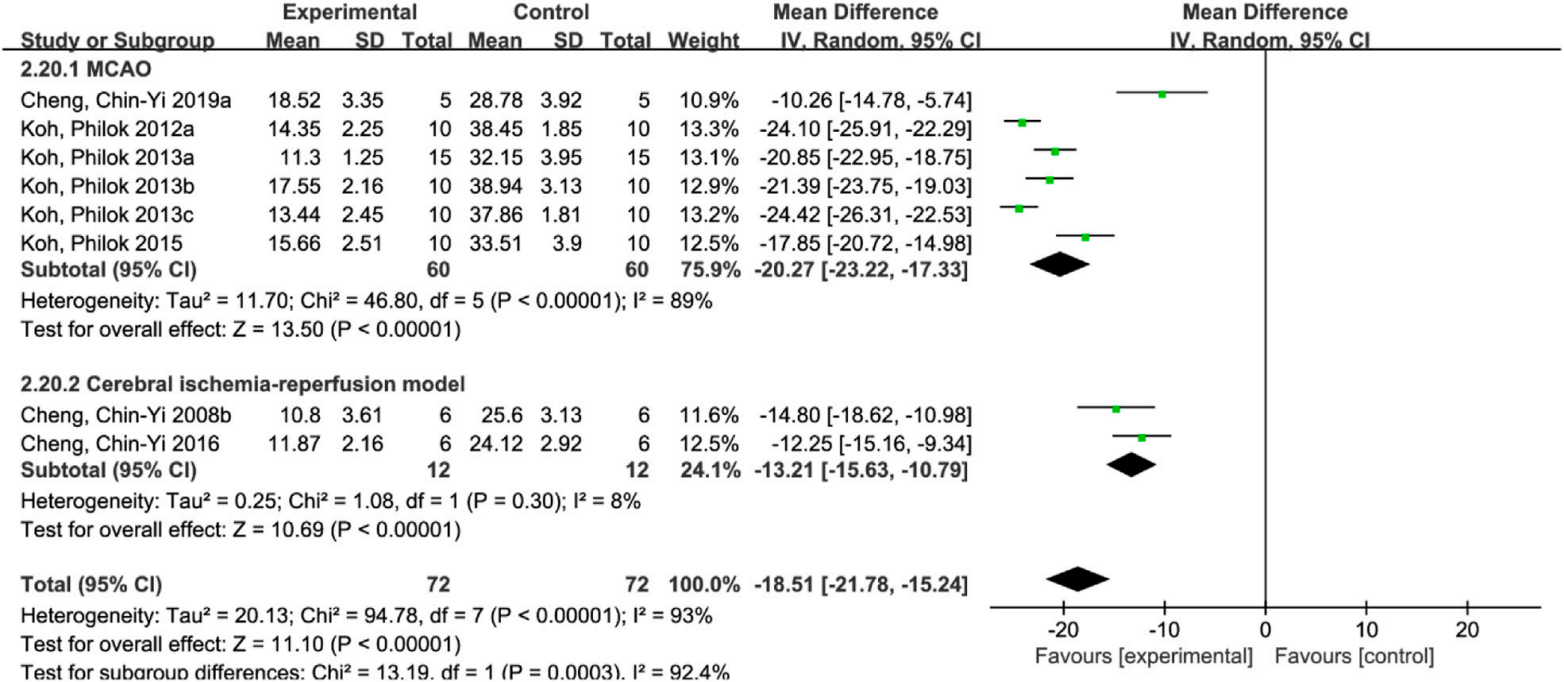
Forest plot of subgroup analysis of infarct size.

Due to the large heterogeneity among the studies (*p* < 0.00001, I^2^ = 93%), subgroup analysis was performed to further analyze the impact of ferulic acid on infarct size. According to different rat models, the studies were divided into permanent ischemic stroke model and ischemic stroke reperfusion model subgroups. The results showed that I^2^ in the subgroup of the permanent ischemic stroke model decreased to 89%, while that in the subgroup of the ischemic stroke reperfusion model decreased to 8%, suggesting that the permanent ischemic stroke model might be the source of heterogeneity. However, the results still support the conclusion that ferulic acid can reduce the infarct size in rat models of ischemic stroke. (Figure 6).

##### 3.4.11.2 Apoptosis index

**FIGURE. 7:**
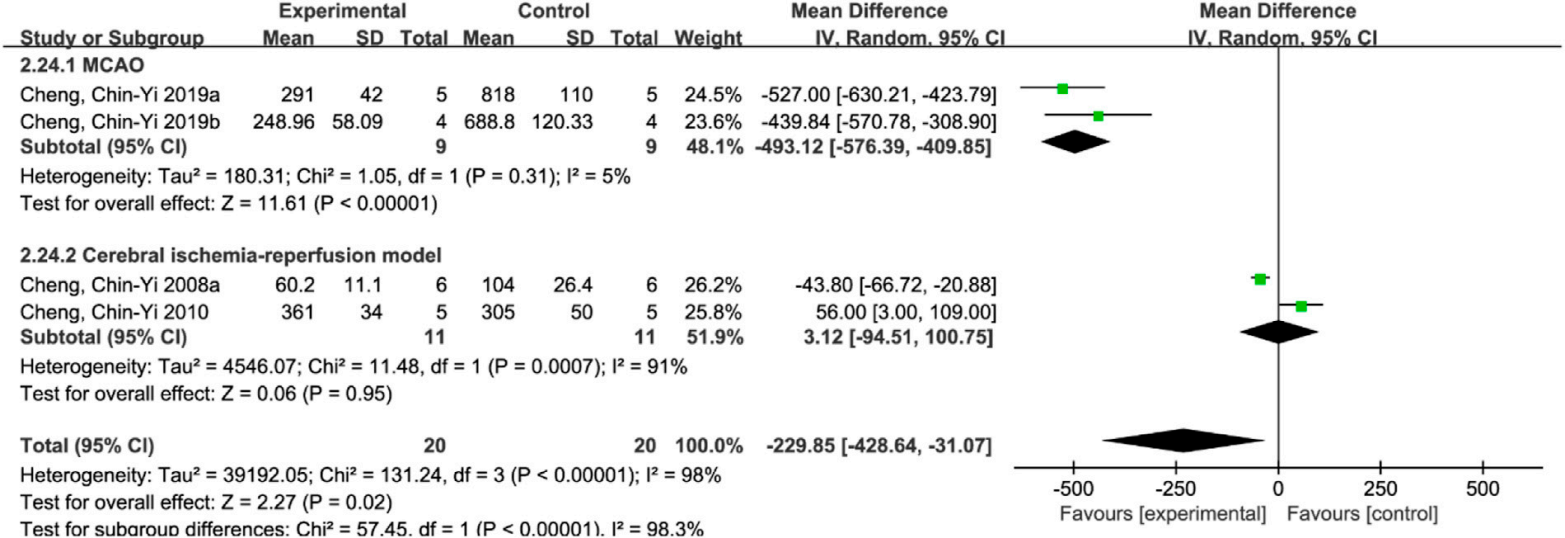
Apoptosis index subgroup analysis forest plot.

Subgroup analyses were performed, stratified by the duration of treatment. The results showed that there was no statistically significant difference between the ferulic acid group and the control group in the treatment duration of 24 h (*p* = 0.32). However, there was a statistically significant difference between the treatment duration of more than 24 h (*p* < 0.00001), but the heterogeneity did not decrease, indicating that the treatment course of ferulic acid in ischemic stroke injury was at least greater than 24 h (Figure 7).

##### 3.4.11.3 Neurological deficit score

**FIGURE 8:**
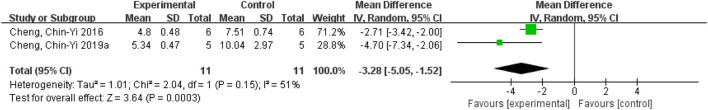
Forest plot of neurological deficit score analysis after Cheng et al. (2019b) was excluded.

After excluding the study reported by Cheng et al. (2019b), the value of I^2^ decreased from 85% to 51%. This suggests that this study may be the main source of heterogeneity. Upon closer examination of the study, it was found that NDS was recorded on the first day after the establishment of ischemic stroke model, while the other two studies recorded NDS on the seventh day after the establishment of ischemic stroke model, suggesting that NDS recorded at different time points might have affected heterogeneity. Nevertheless, the results still support the conclusion that ferulic acid can effectively reduce the neurological deficit score. (Figure 8).

##### 3.4.11.4 Cleaved caspase-3 levels

**FIGURE 9:**
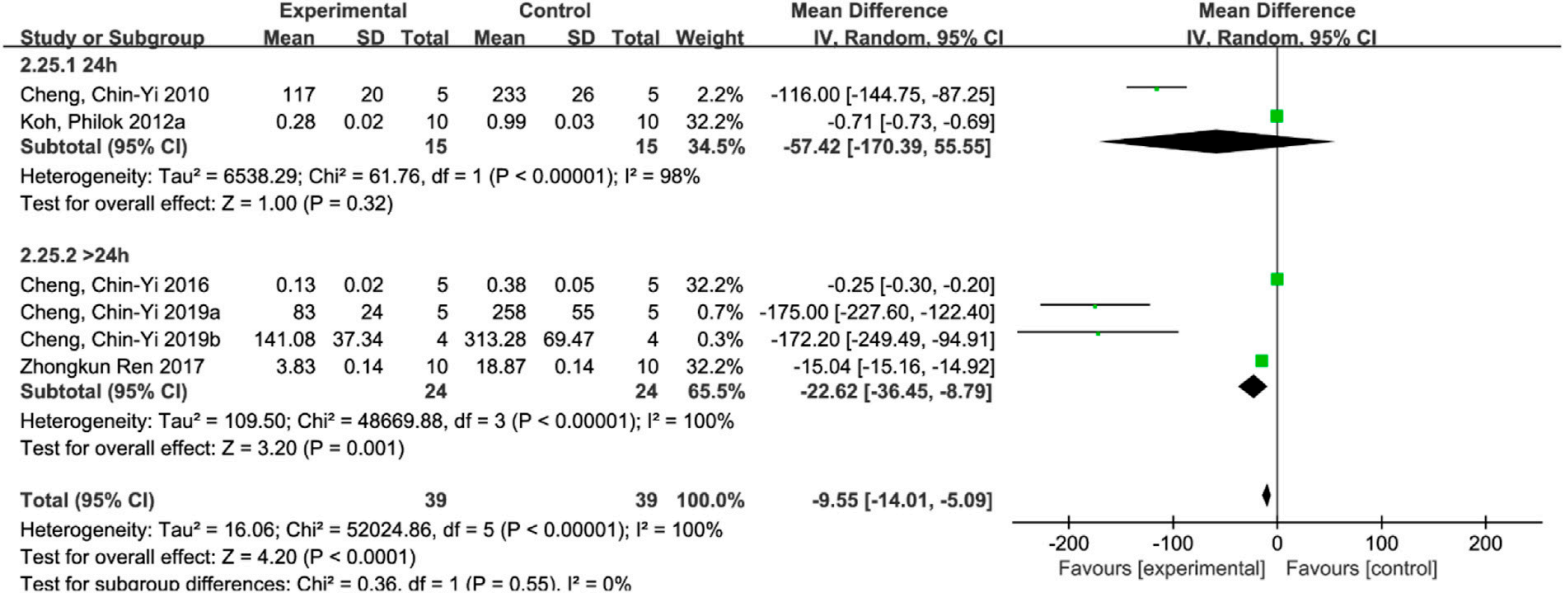
Cleaved caspase-3 level subgroup analysis forest plot.

Subgroup analyses were performed, according to whether the duration of treatment was longer than 24 h. The results showed that there was no statistically significant difference between the ferulic acid group and the control group in the treatment course of 24 h (*p* = 0.32). However, there was a statistically significant difference between the treatment course of more than 24 h (*p* < 0.00001), but the heterogeneity did not decrease, indicating that the treatment course of ferulic acid in ischemic stroke injury was at least greater than 24 h (Figure 9).

##### 3.4.11.5 Mitochondrial Bcl-xL/Bax

**FIGURE 10:**
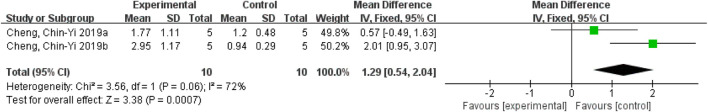
Forest plot of mitochondrial Bcl-xL/Bax analysis after Cheng et al. (2016) exclusion.

After removing Cheng et al. (2016), the heterogeneity increased from 44% to 72%, indicating that this study was the main source of instability. However, the heterogeneity of the remaining two studies decreased to 0%, suggesting that these two studies were considered the main sources of heterogeneity. It was found that Cheng, et al. (2016) used an ischemic stroke-reperfusion model, while the remaining two studies used a permanent ischemic stroke model, suggesting that different rat models might affect the heterogeneity. However, the results still demonstrated that ferulic acid can increase mitochondrial Bcl-xL/Bax ratio in the AIS. (Figure 10).

##### 3.4.11.6 Mitochondrial Bcl-2/Bax

**FIGURE 11:**
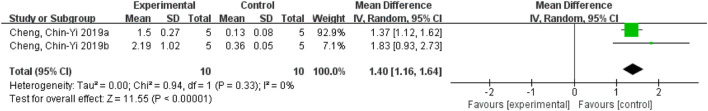
Forest plot of mitochondrial Bcl-2/Bax analysis after Cheng et al. (2016) was excluded.

After removing Cheng et al. (2016) the heterogeneity decreased from 91% to 0%, indicating that this study was the main source of heterogeneity. Due to the small number of studies included, it is speculated that the possible reason is different ischemic stroke models used in the studies. Cheng et al. (2016) used the ischemic stroke reperfusion model, while other studies used the permanent ischemic stroke model. Nevertheless, the results still demonstrated that the ferulic acid group had the effect of increasing mitochondrial Bcl-2/Bax ratio in the ischemic stroke. (Figure 11).

##### 3.4.11.7 Cytochrome C levels

**FIGURE 12:**
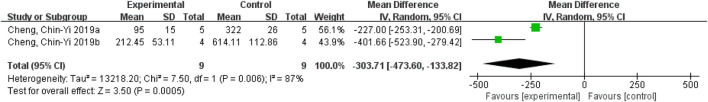
Analysis of forest plots of cytochrome C levels after removing Cheng et al. (2016)

Excluding Cheng et al. (2016) decreased the heterogeneity from 98% to 87%, indicating that this study may be the source of heterogeneity. One study used ischemic stroke-reperfusion model, and the other two studies used permanent ischemic stroke model, suggesting that different models may affect the heterogeneity of the results. Nevertheless, the results still demonstrated that ferulic acid increase cytochrome C levels in the ischemic stroke. (Figure 12).

#### 3.4.12 Publication bias

**FIGURE 13:**
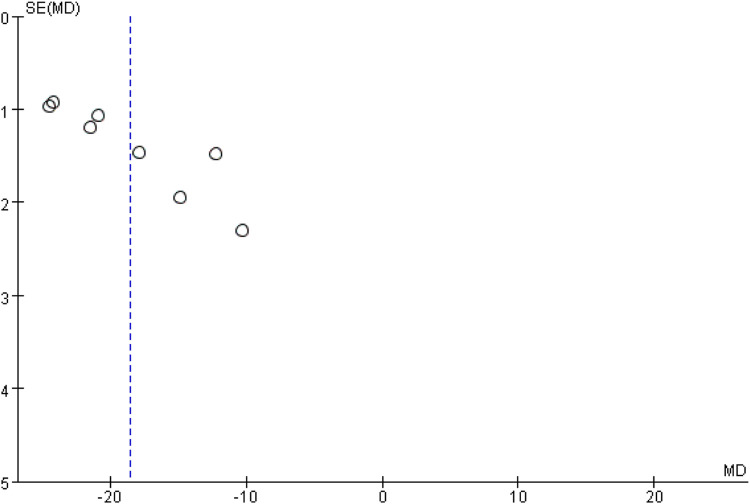
Cerebral infarction volume funnel diagram.

In this study, bias analysis was conducted on the ischemic stroke volume using a funnel plot, as presented in Figure 13. As there were only 8 studies included, the plot was used to assess the potential for publication bias. The asymmetrical distribution of data points along the center line indicates the likelihood of such bias.

## 4 Discussion

Ischemic stroke is a vascular disease caused by the insufficient supply of blood to the brain. Studies have investigated the use of ferulic acid in treating ischemic stroke, there remains a lack of comprehensive systematic evaluation.

This study includes all trials of ferulic acid for AIS injury in rats prior to July 2023. We aimed to comprehensively evaluate the impact of ferulic acid on infarct size, apoptosis index, neurological deficit score, the levels of cleaved caspase-3, phosphorylated Akt, mitochondrial Bcl-xL/Bax, phosphorylated astrocyte PEA-15, hippocampal calcium-binding protein, mitochondrial Bcl-2/Bax, and cytochrome C. The specific analyses are as follows.

(1) First of all, the intervention of ferulic acid can reduce the infarct size and neurological deficit score in rats, indicating that ferulic acid plays an important protective role in rats with ischemic stroke damage.
(2) Furthermore, ischemic stroke injury has been linked to the release of the neurotoxic excitatory neurotransmitter glutamate, which can induce calcium influx and activate calcium-dependent death-signaling proteins, leading to neuronal death (Ted et al., 2014; Zhang and Jiang, 2011). Hippocampal calcium-binding proteins provide an important first line of defense through their ability to buffer calcium overload, allowing the neuron from calcium overload and protecting against calcium-dependent neural damage (Möckel and Fischer, 1994; Fairless et al., 2019). Ferulic acid could significantly increase levels of calcium-binding proteins in the hippocampus, thus inhibiting ischemic stroke-induced calcium increase and protecting the neuron from ischemic stroke injury.
(3) In addition, ischemic stroke injury is closely related to the inflammatory response (Wang and Ni, 2016). The activated astrocytes can release a large number of inflammatory factors to induce the inflammatory response after ischemic stroke. Among which tumor necrosis factor (TNF-α) can be inhibited by phosphorylated PEA-15 (Liu et al., 2017). Ferulic acid can inhibit inflammation by increasing the production of PEA-15, thus conferring the protective effect on ischemic stroke injury.
(4) Last but not least, ischemic stroke injury is also closely associated with cell apoptosis (Peng and Liu, 2017). Normally, the pro-apoptotic factor Bax binds to Bcl-xL by phosphorylated Akt and does not induce apoptosis. When brain cells are in a state of ischemic stress, the phosphorylation of Akt is inhibited, and the expression of negative modulators Bcl-xL and Bcl-2 is reduced, leading to the increase of Bax (Havasi et al., 2008), and the subsequent release of cytochrome C (Zhou and Luo, 2007) from mitochondria, thus activating the signal transduction of apoptosis, caspase-3, and reducing the expression of channel regulatory protein (Jiang et al., 2019). Ferulic acid can promote the phosphorylation of Akt, increase mitochondrial Bcl-xL and Bcl-2 levels, inhibit the expression of Bax, inhibit the release of cytochrome C, reduce cleaved caspase-3 levels, resulting in the inhibition of apoptosis in brain cells.

According to the included literature, the usual dose of ferulic acid in the treatment of ischemic stroke is 56–100 mg/kg. In terms of the time of administration, 14 studies clearly indicated that the drug was administered immediately after the onset of ischemic stroke, 1 study showed that the drug was administered 30 min before ischemic stroke, and 1 study did not explicitly mention the specific time of administration. In addition, behavioral tests were performed 24 h, 3, 5, or 7 days after administration of the drug treatment, or brain tissue was harvested for animal experiments. There is a lack of research on the optimal dose of ferulic acid, the timing of intervention and the duration of medication. The limitations of this study include:

①Among the 16 studies, some studies had inadequate reporting in the aspects of allocation concealment and blind design, which may lead to the risk of bias in selection and measurement. ②Whether ferulic acid can replace alteplase or urokinase, and whether ferulic acid combined with alteplase or urokinase has better efficacy need to be further studied; ③In addition, the optimal dose, course of treatment and timing of intervention also need more trials.

## 5 Conclusion

In summary, this meta-analysis suggests that ferulic acid is effective in the treatment of AIS injury in rats by protecting against ischemia-induced excitatory, inflammatory reactions, and cell apoptosis. Ferulic acid reduces the infarct size and neurological deficit score, and has potential therapeutic value for AIS in humans.

## Data Availability

The original contributions presented in the study are included in the article/Supplementary material, further inquiries can be directed to the corresponding author.
